# Multimodal brain image fusion based on error texture elimination and salient feature detection

**DOI:** 10.3389/fnins.2023.1204263

**Published:** 2023-07-13

**Authors:** Xilai Li, Xiaosong Li

**Affiliations:** School of Physics and Optoelectronic Engineering, Foshan University, Foshan, China

**Keywords:** brain imaging, multimodal brain image fusion, medical assistance, error texture elimination, salient feature detection

## Abstract

As an important clinically oriented information fusion technology, multimodal medical image fusion integrates useful information from different modal images into a comprehensive fused image. Nevertheless, existing methods routinely consider only energy information when fusing low-frequency or base layers, ignoring the fact that useful texture information may exist in pixels with lower energy values. Thus, erroneous textures may be introduced into the fusion results. To resolve this problem, we propose a novel multimodal brain image fusion algorithm based on error texture removal. A two-layer decomposition scheme is first implemented to generate the high- and low-frequency subbands. We propose a salient feature detection operator based on gradient difference and entropy. The proposed operator integrates the gradient difference and amount of information in the high-frequency subbands to effectively identify clearly detailed information. Subsequently, we detect the energy information of the low-frequency subband by utilizing the local phase feature of each pixel as the intensity measurement and using a random walk algorithm to detect the energy information. Finally, we propose a rolling guidance filtering iterative least-squares model to reconstruct the texture information in the low-frequency components. Through extensive experiments, we successfully demonstrate that the proposed algorithm outperforms some state-of-the-art methods. Our source code is publicly available at https://github.com/ixilai/ETEM.

## 1. Introduction

Multimodal brain image fusion (MBIF) (Azam et al., 2022) has important clinical applications, such as tumor segmentation (Zhu et al., 2023), cell classification (Guo et al., 2016), and neurological studies (Catana et al., 2012), and researchers are increasingly drawn to it owing to its ability to utilize multimodal information simultaneously, thereby offering a comprehensive understanding of a particular pathology. The goal of MBIF is to improve image readability and clarity by integrating complementary information from images of different modalities. Positron emission tomography (PET), computed tomography (CT), magnetic resonance imaging (MRI), and single-photon emission CT (SPECT) are typical medical imaging methods that are useful in medical diagnosis. They exhibit unique advantages and inherent drawbacks owing to their different mechanisms.

A plethora of MBIF methods (Kong et al., 2021; Nie et al., 2021) have emerged in the last few years. They can be classified into traditional-based and deep learning (DL)-based techniques. Spatial domain-based (SDB) (Nie et al., 2021) and transform domain-based (TDB) methods (Ullah et al., 2020; Jie et al., 2022; Li et al., 2023) are two types of representative traditional-based methods. TDB methods decompose images into subbands that represent different aspects of the image and design suitable fusion rules based on the distinctive characteristics of each component. Finally, the result is reconstructed by applying the inverse transformation. For instance, Ullah et al. (2020) devised a non-downsampling shear wave transform-based MBIF method. This approach aimed to extract crucial feature information from the source image and enhance the contrast and fidelity of the fusion outcomes. By operating at different scales, from coarse to fine levels, the method can capture significant pixel information. Li et al. (2018) implemented the task of MBIF in the local Laplace transform (LLP) domain to solve the problem of color distortion caused by the fusion process. Furthermore, they introduced generalized intensity-hue-saturation in LLP to ensure the complete transmission of color information. Although TDB methods can obtain good visual effects owing to their multidirectional and multiscale characteristics, they generally have high computational complexity and tend to lose detailed information. SDB methods are used to analyze or calculate the features of the pixels directly in the source image with high computational efficiency. However, these methods are prone to energy loss and residual artifacts during the fusion process, leading to suboptimal fusion results.

Furthermore, some hybrid-based methods (Du et al., 2020; Li et al., 2021a,b; Zhu et al., 2022) were proposed. Li et al. (2021b) proposed a two-layer decomposition model using joint bilateral filtering, which decomposes brain images into an energy layer that contains rich intensity information and a structure layer that reflects structural and detailed information. This model is computationally efficient and can effectively identify useful information. Tan et al. (2021) used multilevel edge-preserving filtering to decompose images into a fine structure, coarse structure, and basic layers, and they achieved a classification of image pixels. Zhu et al. (2022) proposed a hybrid image decomposition model for extracting texture information from source images, taking advantage of the transform and spatial domains. Li et al. (2021) combined dynamic threshold neural P systems with a non-subsampled contourlet transform to develop an MBIF model. However, these decomposition models (Li et al., 2021; Tan et al., 2021; Zhu et al., 2022) may not fully capture the pixel information at different scales and have low computational efficiency when multilayer decomposition is implemented.

DL-based methods (Amin-Naji et al., 2019; Li X. et al., 2020; Xu and Ma, 2021) can roughly be classified into non-end-to-end and end-to-end methods. Typically, non-end-to-end methods (Amin-Naji et al., 2019; Li J. et al., 2020) leverage only the feature extraction capabilities of DL to identify relevant information from disparate source images and create fusion weight maps based on the extracted features. Additionally, end-to-end methods (Xu and Ma, 2021; Le et al., 2022; Ma et al., 2022; Xu et al., 2022) generally perform fusion in an unsupervised manner; they may dispense with the need for brain image datasets in parameter debugging, potentially resulting in a loss of detailed information within the fusion results. Furthermore, the fusion problem is solved by subjectively defining the features of the fusion process, but this may also lead to the distortion of some useful information (Xu and Ma, 2021). To address this problem, Xu and Ma (2021) proposed a brain image fusion network that can preserve chromaticity information and texture details in source images. Conventional DL-based methods often lack consideration of inter-scale information and input source images into a single network, resulting in the potential loss of crucial details. To address this limitation, Tang et al. (2022) proposed an MBIF based on multiscale adaptive transformers. Fu et al. (2021) introduced a novel multiscale residual pyramidal attention network to capture multiscale information in images. This network combines the strengths of both residual attention and pyramidal attention networks, resulting in enhanced performance for MBIF tasks. Wang C. et al. (2022) proposed an unsupervised information gate network for MBIF that can control the contribution of each encoder feature level to the decoder. Additionally, a multiscale cross-attention module was designed to extract salient information at different scales of the source image. Although deep learning has excellent fusion performance in the MBIF domain, the lack of high-quality brain image datasets and over-reliance on the design of loss functions impose significant limitations on these methods. Moreover, DL-based methods typically rely on convolutional operations and can identify locally significant information effectively, but they may be limited in their ability to retain global information.

Recently, some image three-layer decomposition fusion models (Du et al., 2020; Li et al., 2021a) have also emerged. Li et al. (2021a) proposed a three-layer decomposition model based on sparse representation, in which interval gradient filtering was used to decompose the low-frequency layer. Du et al. (2020) used local polar and low-pass filters to decompose each input image into smooth, texture, and edge layers. Although these models could improve the finer classification of pixels, there is room for improvement in their ability to differentiate between basic and intricate information, and the issue of filter selection remains a significant challenge.

In summary, several existing methods (Li et al., 2021a,b) only consider the energy of the pixels when designing fusion rules for the low-frequency components. Some useful texture information may be distributed in the low-frequency components with lower pixel values. The cost of ignoring this useful pixel information is that false textures are introduced in the fusion results, affecting a physician's clinical diagnosis.

In this study, we propose an error texture elimination strategy. During the fusion of low-frequency components, we prioritize designing fusion rules based on pixel energy to preserve the useful energy information from the source images. Because not all texture information in the low-frequency components is useful, the erroneous textures should be removed during texture information fusion. Iterative least squares (ILS) is a recently developed technique. It can exhibit competing edge-preserving smoothing capabilities with limited iterations (Liu et al., 2020b). In this study, we combine rolling guidance filtering (RGF) with ILS and propose a novel image smoothing model, RGF-ILS. The proposed RGF-ILS can effectively filter out texture information from the fused low-frequency components and re-extract the texture in the low-frequency images for fusion. Moreover, considering variations in gradient values and image information in high-frequency components, we propose a salient feature extraction operator that leverages gradient differences and entropy measures. By using this operator, we can effectively identify and retain the most representative detailed textures in the image, resulting in a high-quality fused image. This study's primary contributions are as follows:

1. We propose an MBIF algorithm based on error texture elimination that can effectively retain useful information while eliminating the error texture in source images.
2. We propose an image smoothing model, RGF-ILS, which can effectively separate the energy layer in the fused low-frequency image; the error texture is obtained by subtracting the energy layer from the source images.
3. We propose a significant feature extraction operator based on the gradient difference and entropy that can identify clear high-frequency details and capture and use the global information in the high-frequency components effectively.

## 2. Related works

### 2.1. Local phase coherence

Blurred images can be interpreted as a loss of local phase coherence (LPC), and LPC intensity can be used as an indicator of image clarity. Hassen et al. (2013) developed an LPC-based algorithm for image sharpness measurement that can effectively detect large variations in the sharpness of an image. A given sharpness-evaluated image is passed through a series of log-Gabor filters with scale *N* and orientation *M*. Let *C*_*s,o,k*_ be the complex coefficient at the *o*-th orientation, *s*-th scale, and *k*-th spatial location. The LPC strength at the *k*-th location and *o*-th orientation can be computed as follows:


(1) $$S_{LPC}^{\left\{j,k\right\}}=\frac{R\left\{\prod_{s=1}^{N}C_{s,o,k}^{\omega_{s}}\right\}}{\left|\prod_{s=1}^{N}C_{s,o,k}^{\omega_{s}}\;\right|}$$

where the real part of a complex number is denoted by *R*{·}. The weights are determined based on the magnitude of the first scale factor, *C*_1,*o,k*_, such that directions with higher energy are assigned greater weights.


(2) $$S_{LPC}^{\left\{k\right\}}=\frac{\sum_{o=1}^{M}\left|C_{1,o,k}\right|S_{LPC}^{\left\{o,k\right\}}}{\sum_{o=1}^{M}\left|C_{1,o,k}\right|+V}$$

where *V* is a constant. The set of $S_{LPC}^{\left\{k\right\}}$ values obtained at all locations form a spatial LPC map that reflects the information of pixels with significant sharpness variations in the input image. Give an input image, *f*, we denote the operation of obtaining LPC maps from LPC intensity measurements as


(3) $$\begin{array}{l} SFP=LPC\left(f\right) \end{array}$$

where *SFP* represents the output salient feature map, *LPC*(·) is the obtained LPC intensity measurement operation, and f is the input source image. Refer to Hassen et al. (2013) for further information regarding LPC.

### 2.2. ILS

Edge-preserving filters (Mo et al., 2021; Yao et al., 2022; Zhang and He, 2022) have attracted increasing attention in the field of image processing in recent years because they can preserve different hierarchies while smoothing images. As edges generally contain important image information and represent large differences between the local pixels of an image, the main purpose of an edge-preserving filter is to preserve high-contrast edges and remove low-contrast or subtle variations in the edge detail. The details and texture information are extremely important for the description of lesions in brain image fusion, and the introduction of errors or omission of certain textures may cause difficulties in diagnosis. Therefore, the effective extraction of details and texture information from brain images is an important task.

Liu et al. (2020b) proposed an image smoothing filter based on ILS that can effectively achieve image smoothing and edge preservation. The filter involves the minimization of the following objective functions:


(4) $$\begin{array}{l} E\left(u,\;f\right)=\sum_{s}\left({\left(u_{s}\;-\;f_{s}\right)}^{2}\;+\;\sum_{*\in \left\{x,\;y\right\}}\;\phi_{p}\left({\text{∇ }u}_{*,\;s}\right)\right) \end{array}$$

where *u* is the output result, *s* is the pixel position, and ∇u∗(*∈{x,y}) represents the gradient of *u* along the x-axis/y-axis. The penalty function, ϕ_*p*_(·), is defined as


(5) $$\begin{array}{l} \phi_{p}\left(x\right)={\left(x^{2}+\varepsilon \right)}^{\frac{p}{2}} \end{array}$$

Similar to that in Liu et al. (2020b), and constant ε is set to 0.0001 in this study. The norm power, *p*, is set to 0.8 in this study. According to Liu et al. (2020b), (4) can be rewritten as


(6) $$\begin{array}{l} u^{n+1}\\ \;=\underset{u}{\arg\min}\underset{s}{\sum }\left({\left(u_{s}-f_{s}\right)}^{2}+\text{λ}\underset{*\in \left\{x,y\right\}}{\sum }\frac{1}{2}{\left(\sqrt{c}\nabla u_{*,s}-\frac{1}{\sqrt{c}}\mu_{*,s}^{n}\right)}^{2}\right) \end{array}$$

where *c* = *pϵ*^*p*/2^>1 and *n* represents the number of iterations and is set to 3 in this study. Furthermore, $\mu_{*}^{n}{\;}_{,s}$ is the optimal condition, which can be defined as follows:


(7) $$\mu_{*}^{n}{\;}_{,s}=c\nabla \mu_{*}^{n}{\;}_{,s}-p\nabla \mu_{*}^{n}{\;}_{,s}{\left({\left(\nabla \mu_{*}^{n}{\;}_{,s}\right)}^{2}+\varepsilon \right)}^{\frac{p}{2}-1}{,}^{*}\in \left\{x,y\right\}.$$

Each iteration in Eq. (6) is a least-squares problem, and *u* is calculated iteratively; thus, Eq. (6) can be expressed as ILS. We use the Fourier transform (FT) and inverse FT (IFT) to solve Eq. (6), as follows:


(8) $$\begin{array}{l} u^{n+1}\\ \;=F^{-1}\left(\frac{F\left(f\right)+\frac{\text{λ}}{2}\left(F\left(\nabla_{x}\right)\right)\cdot F\left(\mu_{x}^{n}\right)+\left(F\left(\nabla_{y}\right)\right)\cdot F\left(\mu_{y}^{n}\right)}{F\left(1\right)+\frac{c}{2}\cdot \text{λ}\left(F\left(\nabla_{x}\right)\right)\cdot F\left(\mu_{x}^{n}\right)+\left(F\left(\nabla_{y}\right)\right)\cdot F\left(\mu_{y}^{n}\right)}\right), \end{array}$$

where *F*(·) and *F*^−1^(·) represent the Fourier transform (FT) and inverse FT, respectively. *F*(·) denotes the complex conjugate of *F*(·), while *F*(1) denotes the FT of the delta function. Refer to Liu et al. (2020b) for more details on ILS.

## 3. Proposed method

**Figure 1:**
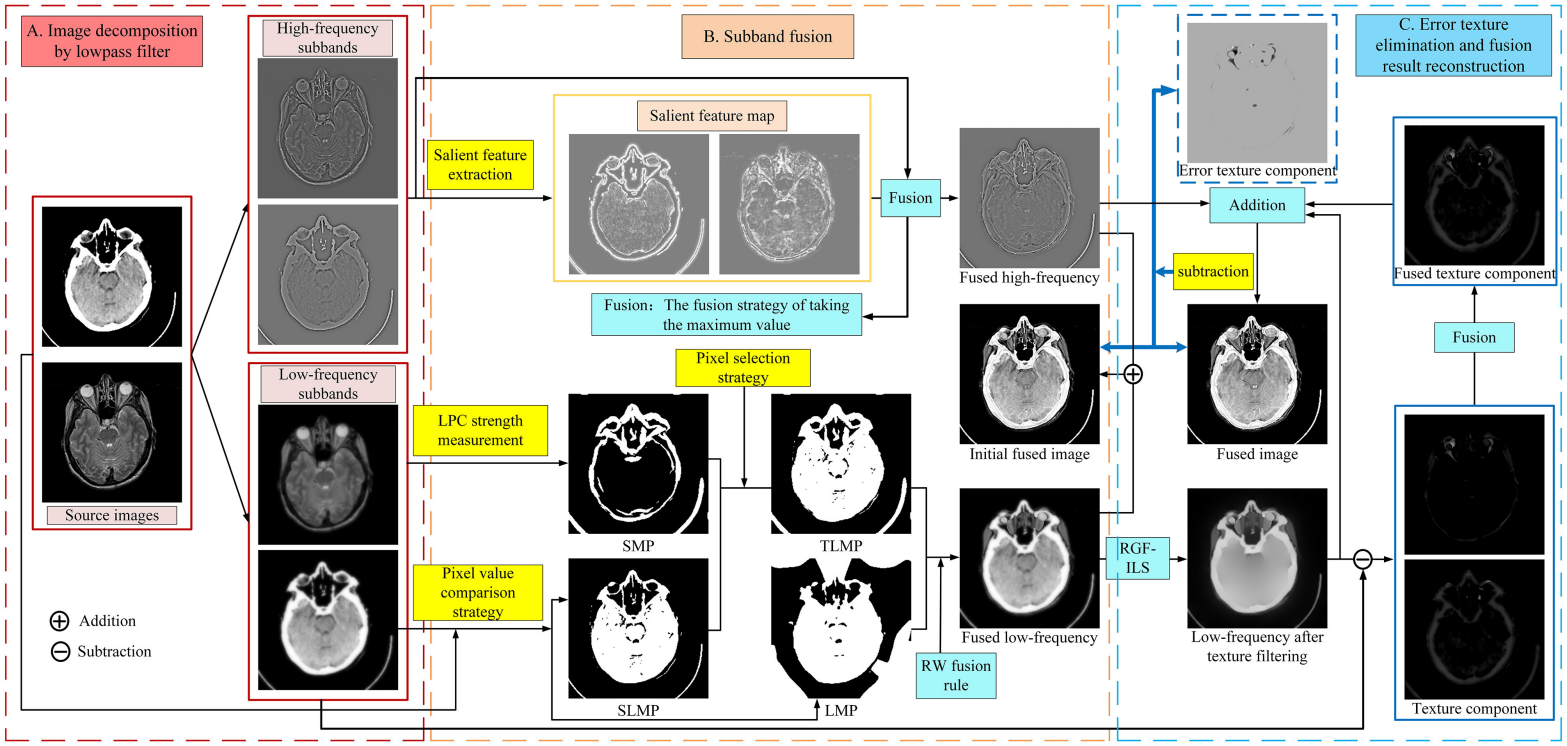
Framework of the proposed method.

The schematic diagram of the proposed MBIF is shown in Figure 1, which involves three steps: image decomposition, subband fusion, and error texture removal and fusion result reconstruction. First, a low-pass filter comprising discrete gradient operators is used to decompose the source image; then, different fusion rules are designed to fuse the different subbands. Moreover, we propose a new saliency measurement operator to effectively detect the significant detailed information in the high-frequency components. After obtaining the low-frequency fusion result, the proposed RGF-ILS model is used to filter the texture information in the fused low-frequency image and re-extract useful texture information to construct the final fusion result. Figure 1 depicts the anatomical brain image fusion example; the anatomical and functional brain image fusion is outlined in Section 3.4.

### 3.1. Image decomposition

We first decompose the source images by solving the following optimization function:


(9) $$\underset{f_{t}^{l}}{argmin}{\left\|f_{t}-f_{t}^{l}\right\|}_{F}^{2}+\beta \left({\left\|g_{a}\;*\;g_{t}^{l}\right\|}_{F}^{2}+{\left\|g_{b}\;*\;g_{t}^{l}\right\|}_{F}^{2}\right),$$

where *f*_*t*_ is the *t*-th source image and $f_{t}^{l}$ represents its low-frequency layer. The operators *g*_*a*_ = [−1, 1] and $g_{b}={\left[-1,1\right]}^{T}$ are the gradient operations in the vertical and horizontal directions, respectively, and * denotes the convolution operation. Our study adopts a regularization parameter, *β*, value of 3. Equation (9) can be expressed as a Tikhonov regularization term. Because it relies solely on the F-parameter, this objective function can be efficiently solved in the discrete Fourier domain. Next, the high-frequency components are obtained by applying the following formula:


(10) $$\begin{array}{l} f_{t}^{h}=f_{t}-f_{t}^{l}. \end{array}$$

### 3.2. Subband fusion

**Figure 2:**
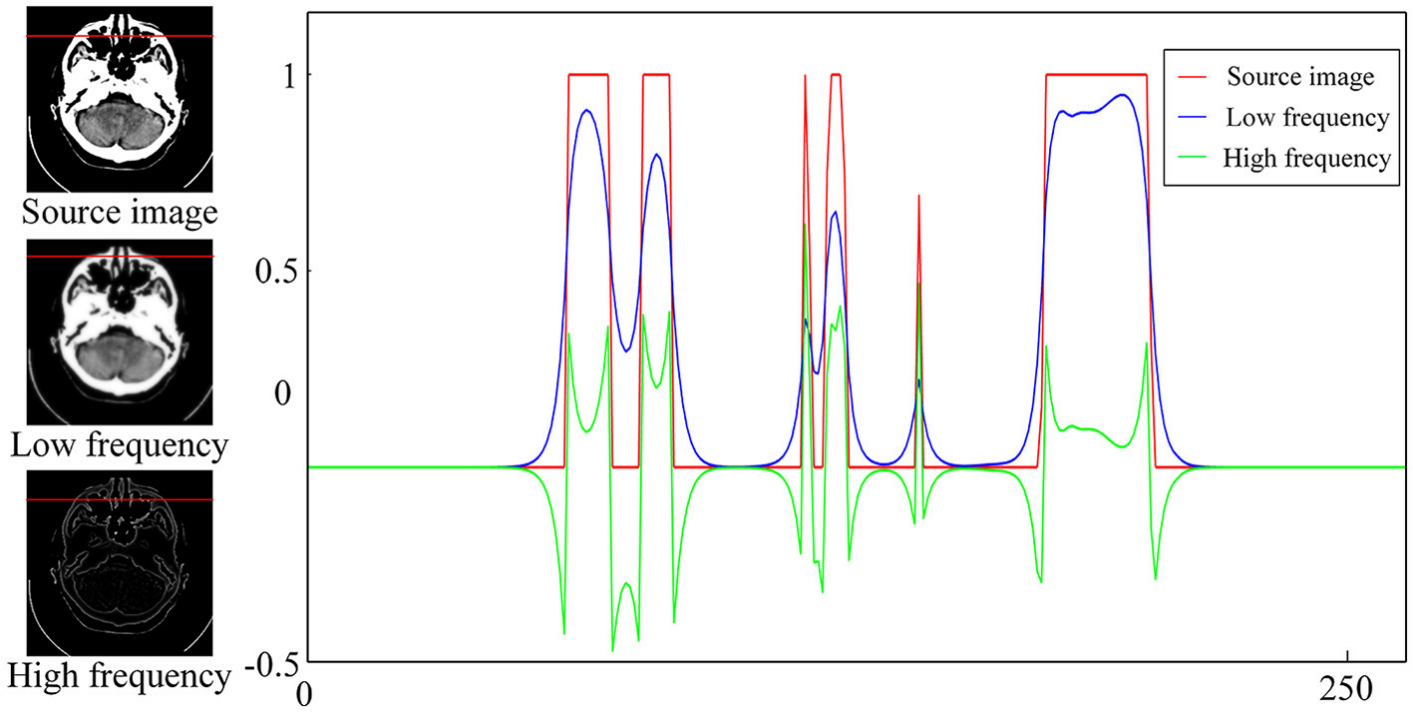
Intensity changes in the source image and the corresponding high- and low-frequency components.

Figure 2 depicts the normalized intensity changes in different image components. Specifically, the low-frequency subband exhibits a slow change in the pixel intensity and is smoother compared with the source image but can maintain the most intensity information. By contrast, the high-frequency subband represents the detailed information, corresponding to the part where the pixel intensity values change faster and the pixel intensity values are smaller. Therefore, different fusion rules should be designed for different subbands to effectively retain useful information. If inappropriate fusion rules are adopted, residual artifacts, an excessive contrast level, and color distortion will appear in the fusion results. In this study, we propose different rules to fuse the subbands according to their respective characteristics. The specific process is outlined as follows.

#### 3.2.1. High-frequency subband fusion

The parts of the source image with more drastic gradient value changes correspond to the clear details of the image, whereas the entropy of the image reflects the amount of information that is contained therein. When the number of clear details in the high-frequency components is high, the entropy value is large.

Therefore, inspired by Tai and Brown (2009), a new fusion rule based on the gradient difference and entropy are proposed to capture the clear details and fuse the high-frequency subbands, as follows:


(11) $$\begin{array}{l} GE_{t}\left(i,j\right)\\ \;=\frac{\max\left|\nabla f_{t}^{h}(i^{'},j^{'})\right|\times C_{t}}{\max\left\{\max\left|\nabla f_{t}^{h}(i^{'},j^{'})\right|\right\}-\min\left\{\min\left|\nabla f_{t}^{h}(i^{'},j^{'})\right|\right\}}, \end{array}$$

where (*i*′, *j*′) ∈ ℕ(*x, y*) is the neighborhood of (*i, j*); we set ℕ(*x, y*) to be a local window of size *ψ* × *ψ*, and *C*_*t*_ is expressed as


(12) $$C_{t}=\left(\max f_{t}(i^{\prime },j^{\prime })-\min f_{t}(i^{\prime },j^{\prime })\right)\times e^{0.3\times E_{\max}}{\;}^{\left|\nabla f_{t}^{h}(i^{\prime },j^{\prime })\right|,}$$

where $E_{\max}\left|\nabla f_{t}^{h}(i^{\prime },j^{\prime })\right|$ denotes the entropy of $\left|\nabla f_{t}^{h}(i^{\prime },j^{\prime })\right|$. In this manner, when numerous clear details are contained in the high-frequency components, the entropy is high, and a large weight is obtained in the *GE*_*t*_. Finally, the high-frequency fusion result is obtained after determining the decision map of the high-frequency components by comparing the pixel value size:


(13) $$\begin{array}{l} FH=\overset{T}{\underset{t}{\sum }}f_{t}^{h}\times HMP_{t} \end{array}$$

where


(14) $$HMP_{t}\left(i,j\right)=\left\{ \begin{array}{l} \;1,if\underset{t}{argmax}\left\{GE_{1}\left(i,j\right),GE_{2}\left(i,j\right),...,GE_{t}\left(i,j\right),...\right\}\\ 0,\;otherwise. \end{array}\right.$$

#### 3.2.2. Low-frequency subband fusion

##### 3.2.2.1. Initial decision map acquisition

The energy information of an image, such as its brightness and contrast, is generally concentrated in the low-frequency subbands. In general, pixel points with higher energy have larger pixel values. Therefore, a preliminary low-frequency fusion decision map (LMP) can be produced by comparing pixel values across various low-frequency subbands:


(15) $$LMP_{t}=\left\{ \begin{array}{l} 1,if\underset{t}{argmax}\left\{f_{1}^{l},f_{2}^{l},...,f_{t}^{l},...\right\}\\ 0,\;otherwise. \end{array}\right.$$

The output of the low-pass filter typically results in a blurred and smooth image. This blurring can be interpreted as a reduction in high-frequency energy, a reduction in contrast, or an extension of the edge width. As such, the blurred visual perception can be considered a loss of LPC. Therefore, LPC intensity can be introduced as a measure of significant information in the image (Hassen et al., 2013). Consequently, we introduce an image sharpness evaluation algorithm based on LPC to extract the brightness information in low-frequency images, as follows:


(16) $$\begin{array}{l} SFP_{t}=LPC\left(f_{t}^{l}\right). \end{array}$$

Then, decision map SMP is obtained by comparing the pixel value size:


(17) $$SMP_{t}=\left\{ \begin{array}{l} 1,if\underset{t}{argmax}\left\{SFP_{1},SFP_{2},...,SFP_{t},...\right\}\\ 0,\;otherwise. \end{array}\right.$$

As the LPC-based algorithm can only identify the pixel points with a large variation in sharpness, the remaining valid pixel points are obtained by comparing the sizes of the pixel values in the source image and generating decision map SLMP:


(18) $$SLMP_{t}=\left\{ \begin{array}{l} \text{​}1,if\underset{t}{argmax}\left\{f_{1},f_{2},...,f_{t},...\right\}\\ 0,\;otherwise. \end{array}\right.$$

Finally, we propose a pixel selection rule to integrate the significant pixel points that are identified in SMP and SLMP to obtain a decision map TLMP.


(19) $$TLMP_{t}=\left\{ \begin{array}{l} 1,SLMP_{t}\left(i,j\right)=1\;and\;SMP_{t}\left(i,j\right)=0\\ SLMP\left(i,j\right),\;otherwise. \end{array}\right.$$

##### 3.2.2.2. Final decision map acquisition

*Step 1. Image smoothing*

A guided filter (GF) is used to smooth the LMP and TLMP obtained earlier.


(20) $$\begin{array}{l} GMP_{A}=GF\left(f_{1},LMP_{1},r,\varsigma \right) \end{array}$$

(21) $$\begin{array}{l} GMP_{B}=GF\left(f_{1},TLMP_{1},r,\varsigma \right), \end{array}$$

where *GF*(·) denotes the guided filtering operation (Sasidharan et al., 2015), *GMP*_*m*_ represents the output of the guided filter, and *r* and ς indicate the spatial and range weights and are set to 5 and 0.3, respectively.

*Step 2. Decision map optimization*

After *GMP*_*A*_ and *GMP*_*B*_are obtained, the random walk (RW) (Yao et al., 2022) is used to combine the two decision maps to generate the final low-frequency decision map (i.e., to extract the representative energy feature information in the low-frequency subbands so that robust fusion can be achieved).

To begin, weights for the edges connecting the node *x*_*i*_ and seed *S*_*k*_ (denoted as *y*_*ik*_) and for the edges connecting adjacent nodes *x*_*i*_ and *x*_*j*_ (denoted as ω_*ij*_) need to be assigned. Similar to Grady (2006), we set ω_*ij*_ to


(22) $$\begin{array}{l} \omega_{ij}=exp\left(-\frac{{\left(g_{i}-g_{j}\right)}^{2}}{\sigma^{2}}\right),\;g=f_{1} \end{array}$$

where *g* is the source image *f*_1_, and *σ* is set to 0.1 in this study. Furthermore, *y*_*im*_ can be defined as


(23) $$y_{im}=\left\{ \begin{array}{l} GMP_{m}^{i},\;GMP_{m}^{i}>0.8\\ 1-GMP_{m}^{i},\;GMP_{m}^{i}<0.2\\ \;0,\;otherwise,\; \end{array}\right.$$

where *m* = {*A, B*} and $GMP_{m}^{i}$ denotes the intensity value of pixel *i* in *GMP*_*m*_. According to the foregoing analysis, pixels with the largest or smallest intensity values are more likely to be selected for the final decision map. Therefore, we set $y_{im}=GMP_{m}^{i}$ when $GMP_{m}^{i}$ is greater than 0.8 (*GMP*_*m*_ is normalized to [0,1]), $y_{im}=1-GMP_{m}^{i}$ when $GMP_{m}^{i}$ is <0.2, and *y*_*im*_ = 0 when $GMP_{m}^{i}$ is between 0.2 and 0.8.

According to Grady (2005), Shen et al. (2011), and Ma et al. (2017), to obtain the unknown probabilities $u_{X}^{m}$, it is necessary to minimize the energy function given below:


(24) $$\begin{array}{l} J^{m}={\left(u^{m}\right)}^{T}Lu^{m}. \end{array}$$

According to Grady (2005), solving for the probability of reaching the seed *S*_*A*_ for the first time and determining whether the estimated decision map based on the estimated probability is possible:


(25) $$M\left(x_{i}\right)=\left\{ \begin{array}{l} GMP_{A}\left(x_{i}\right),\;u_{X}^{A}\left(x_{i}\right)>0.5\\ \;GMP_{B}\left(x_{i}\right),\;otherwise. \end{array}\right.$$

*Step 3. Final decision map acquisition*

Finally, by evaluating the consistency between the center pixel and surrounding pixels, we can delete the incorrect pixels to obtain the final low-frequency fusion decision map, *FLMP*:


(26) $$FLMP\left(i,j\right)=\left\{ \begin{array}{l} \;1,\;if\;\sum_{\left(a,b\right)\in \Phi }FM\left(i+a,j+b\right)\geq \frac{\Phi }{2}\\ 0,\;otherwise, \end{array}\right.$$

where Φ is a square field centered at (*i, j*) with size 9 × 9. Then we can construct the fused low-frequency subband, *FL*, based on the final low-frequency decision map, *FLMP*:


(27) $$\begin{array}{l} FL=f_{1}^{l}\times FLMP+f_{2}^{l}\times \left(1-FLMP\right). \end{array}$$

Although the low-frequency fusion results that are obtained at this point can effectively retain the useful information in the source image, a small fraction of useful image textures may appear in places where the pixels are not very active and are overlooked during the fusion process, thereby introducing erroneous texture information. A small part of the texture may form an important basis for doctors' diagnoses of brain images. Thus, the texture information in the *FL* is reconstructed to remove the pixel information that is misjudged as correct.

### 3.3. Error texture removal and reconstruction of fusion results

In the fusion of low-frequency subbands, popular brain image fusion algorithms (Li et al., 2021b; Huang et al., 2022; Wang G. et al., 2022; Zhang et al., 2022) prioritize retaining as much energy information as possible in the low-frequency components, often disregarding the residual texture information present in these components. Furthermore, the texture and detailed information are fused with the energy information. However, certain algorithms (Li et al., 2021b; Zhang et al., 2022) design fusion rules for the low-frequency subbands based on energy or contrast and do not specifically target the residual texture details therein. Therefore, some incorrect textures may appear in the low-frequency component fusion, and some useful information may be lost, resulting in reduced image contrast and sharpness.

We propose an error texture removal strategy based on RGF-ILS to solve the aforementioned problems and avoid the influence of error texture information on the fusion results. This model can filter the texture information present in the low-frequency components of the fusion without affecting the distribution of the energy information.

#### 3.3.1. Error texture removal

##### 3.3.1.1. Proposed RGF-ILS

Liu et al. (2020a) developed an image smoothing filter for edge preservation by embedding edge retention filtering into the least-squares model. The RGF (Qi et al., 2014) can effectively remove the gradients with small changes in the gradient map and retain the large gradients that reflect significant pixel changes. Furthermore, the RGF can automatically refine the edges with a rolling mechanism to retain large-scale structural information optimally compared with the edge-preserving filter that was used in Liu et al. (2020a). Inspired by Liu et al. (2020a), we propose the RGF-ILS model to effectively combine the advantages of edge-preserving filters with those of the ILS model presented earlier. Letting *p* and *q* index the pixel coordinates in the image, we express the RGF as


(28) $$\begin{array}{l} J^{\eta +1}\left(p\right)\;\\ \text{​​​​​​​​​​​​​​​​​​​​​​}=\frac{1}{K_{p}}\sum_{q\in N\left(p\right)}\exp\left(-\frac{{\left\|p-q\right\|}^{2}}{2\sigma_{s}^{2}}-\frac{\left\|J^{\eta }\left(p\right)-J^{\eta }\left(q\right)\right\|}{2\sigma_{r}^{2}}\right)I\left(q\right), \end{array}$$

where


(29) $$\begin{array}{l} K_{p}=\underset{q\in N\left(p\right)}{\sum }\exp\left(-\frac{||p-q|{|}^{2}}{2\sigma_{s}^{2}}-\frac{||J^{\eta }\left(p\right)-J^{\eta }\left(q\right)||}{2\sigma_{r}^{2}}\right), \end{array}$$

in which *J*^*η*+1^(*p*) is the result in the *η*-th iteration, and *η* is the number of iterations and is set to 2 in this study. Moreover, *I* is the input image, and *σ*_*s*_ and *σ*_*r*_ control the spatial and range weights and are set to 10 and 0.008, respectively. *N*(*p*) is the set of neighboring pixels of *p*. In this study, *F*_*RGF*_(*I*, *σ*_*s*_, *σ*_*r*_, *η*) is used to represent the RGF operation. Subsequently, we embed the RGF into the ILS model and rewrite Eq. (7) as


(30) $$\mu_{*}^{n}{\;}_{,s}=c\nabla \mu_{*}^{n}{\;}_{,s}-p\nabla \mu_{*}^{n}{\;}_{,s}{\left({\left(\nabla \mu_{*}^{n}{\;}_{,s}\right)}^{2}+\varepsilon \right)}^{\frac{p}{2}-1}{,}^{*}\in \left\{x,y\right\}.$$

where


(31) $$H_{*}^{n}{\;}_{,s}=F_{RGF}\left(u_{*}^{n}{\;}_{,s}\sigma_{s},\sigma_{r},\eta \right).$$

**Figure 3:**
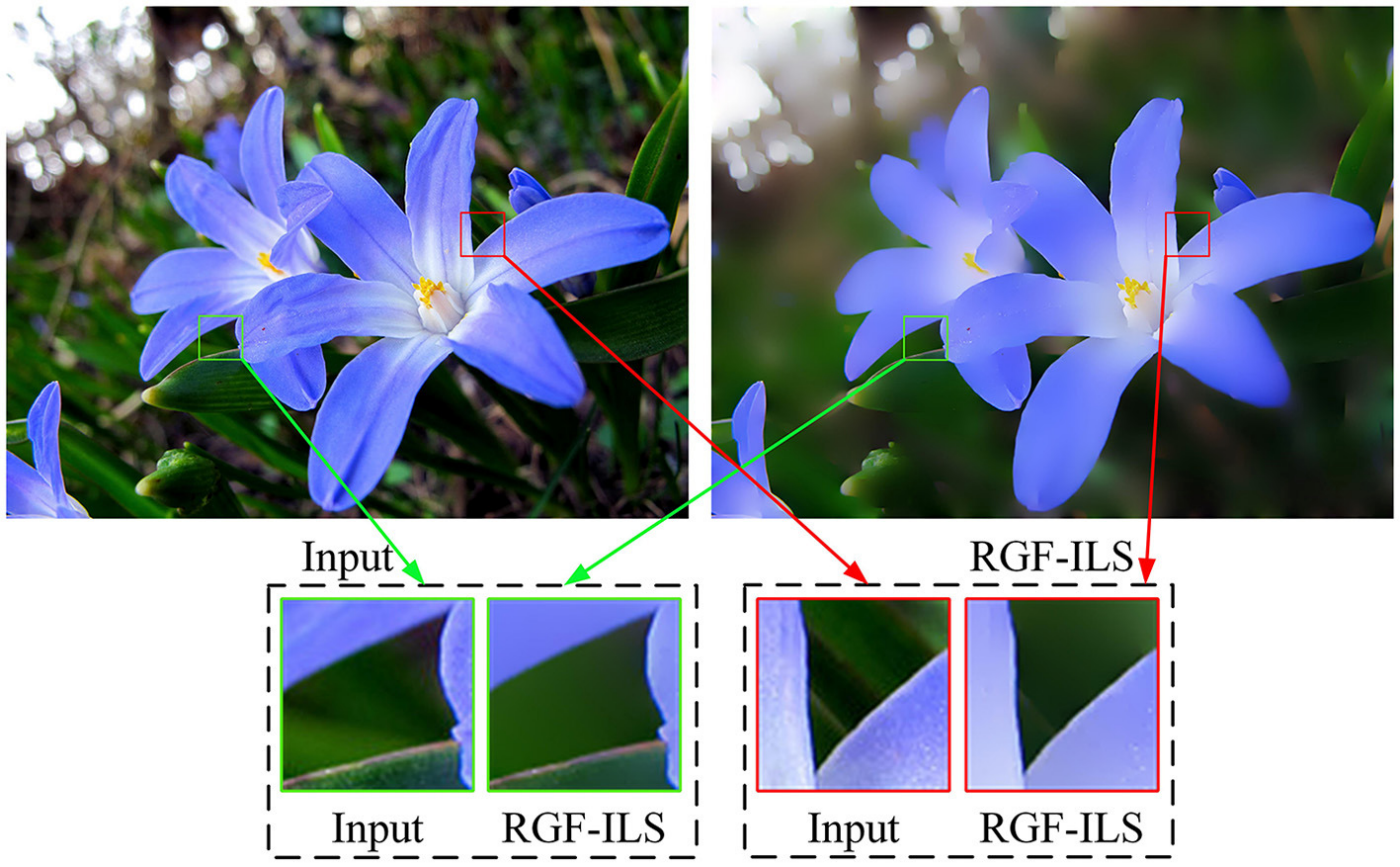
Smoothing example of the proposed RGF-ILS model. The input image is on the **(left)**, and the output image is on the **(right)**.

As illustrated in Figure 3, the proposed RGF-ILS model can effectively smooth the texture and details in the image and retain the edges with significant changes.

##### 3.3.1.2. Texture information reconstruction

First, we use the RGF-ILS model to filter out the texture information in the *FL* and obtain the filtered low-frequency subband, FL.

Subsequently, the difference between the low-frequency subband, $f_{t}^{l},$ and FL is determined to extract the texture information:


(32) $$\begin{array}{l} TE_{t}=f_{t}^{l}-FL. \end{array}$$

At this point, not all the acquired texture information, *TE*_*t*_, is useful. Thus, we use the pixel values of the different texture maps to consider whether the texture information is required, i.e., to compare the pixel value size for obtaining the texture decision map, *TMP*:


(33) $$TMP_{t}=\left\{ \begin{array}{l} \;1,if\underset{t}{argmax}\left\{TE_{1},TE_{2},...,TE_{t},...\right\}\\ 0,\;otherwise. \end{array}\right.$$

The final fused texture component, denoted as *FT*, can be obtained based on the *TMP*:


(34) $$\begin{array}{l} FT=TE_{1}\times TMP+TE_{2}\times \left(1-TMP\right). \end{array}$$

#### 3.3.2. Reconstruction of fusion results

After obtaining the high-frequency fusion result, *FH*, texture fusion result, *FT*, and filtered low-frequency component, FL, we can reconstruct the final fusion result *F*:


(35) $$\begin{array}{l} F=FH+FT+FL. \end{array}$$

**Figure 4:**
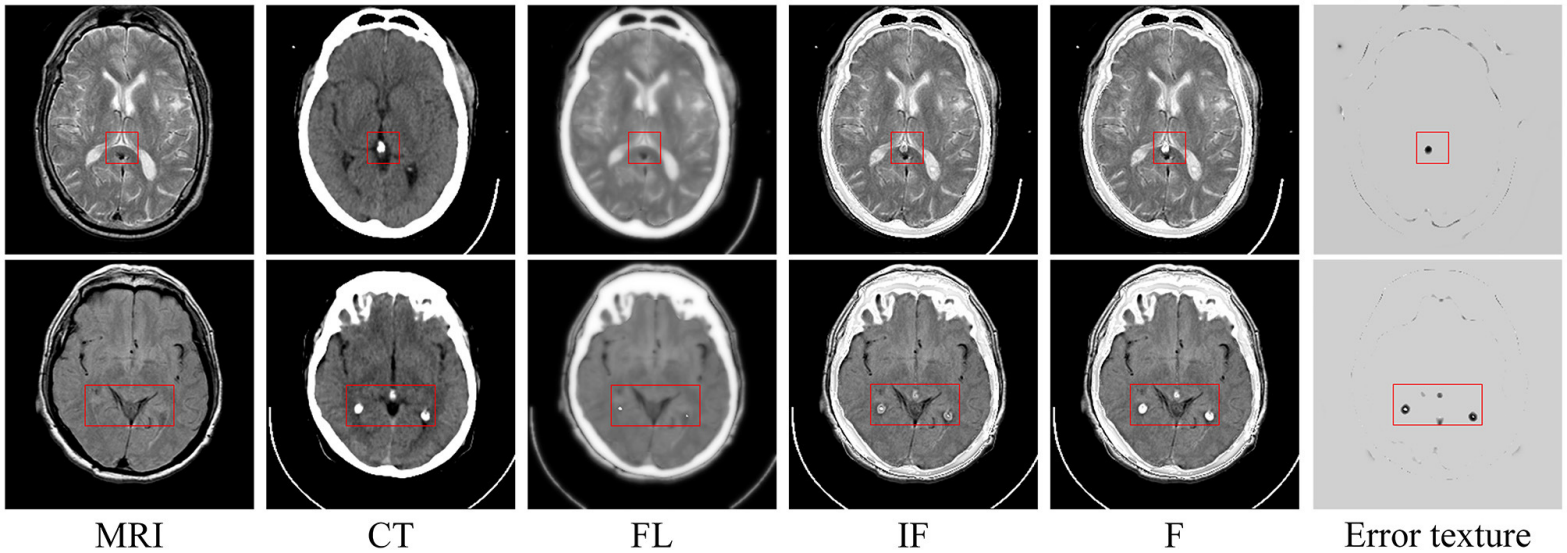
Experimental results of error texture removal strategy. **Left to right**: MRI image, CT image, fused low-frequency subbands, initial fusion results, final fusion results, and error texture frequency.

As illustrated in Figure 4, if the *FL* and *FH* are summed to obtain the initial fusion result, *IF*, that is,


(36) $$\begin{array}{l} IF=FL+FH. \end{array}$$

The fusion results may lead to a loss of some useful detailed information. The red boxes in the last column in Figure 4 contain incorrect texture information, and a decrease in contrast and loss of energy information occurs. The proposed method effectively eliminates error texture, restores image contrast, and achieves higher-quality fusion results [see Figure 4 (F)].

#### 3.4. Fusion of anatomical and functional brain images

For anatomical and functional image fusion tasks, we convert color images into YUV color space, where Y channels represent brightness and U and V channels describe color and saturation, respectively.

## 4. Experiments

Extensive experimental analyses and comparisons are conducted to verify the effectiveness of the proposed algorithm. In the following sections, we abbreviate the proposed algorithm as ETEM.

### 4.1. Experimental setup

#### 4.1.1. Comparison methods

Nine representative state-of-the-art methods are compared in our experiment, which are as follows: TDSR (Li et al., 2021a), MLMG (Tan et al., 2021), JFBM (Li et al., 2021b), LRD (Li X. et al., 2020), U2Fusion (Xu et al., 2022), EMFusion (Xu and Ma, 2021), MATR (Tang et al., 2022), MSRPAN (Fu et al., 2021), and SwinFusion (Ma et al., 2022). Among them, U2Fusion and SwinFusion are general methods; the remaining comparison methods are designed for brain image fusion, thereby making the comparison experiment targeted and fair. Moreover, the parameters of all comparison methods are set exactly as recommended in the relevant literature.

#### 4.1.2. Dataset and experimental platform

The popular publicly available dataset from the Harvard Medical School database^1^ is used as the dataset; it contains 300 sets of aligned multimodal brain images. These source images cover three multimodal brain image fusion tasks: CT-MRI, PET-MRI, and SPECT-MRI.

The experiment for testing the proposed method and nine comparison methods is conducted on a computer equipped with an AMD Ryzen 5 4600H Radeon graphics processor and an NVIDIA GeForce GTX 1650 graphics card.

#### 4.1.3. Evaluation metrics

Eight objective evaluation metrics are used to comprehensively evaluate the quality of the experimental results. The metrics are the normalized mutual information (Q_MI_), Piella metric (Q_S_), Chen–Blum metric (Q_CB_), non-linear correlation information entropy (Q_NCIE_) (Liu et al., 2012), average gradient (AG) (Cui et al., 2015), structural similarity index (SSIM) (Wang et al., 2004), spatial frequency (SF) (Zheng et al., 2007), and peak signal-to-noise ratio (PSNR) (Zhang, 2022). Q_MI_ measures the mutual information between the source and fused images. Q_NCIE_ evaluates the retained non-linear correlation information entropy in the fused image. Q_CB_ can evaluate the fused image from the perspective of visual salience. The SSIM metric evaluates the similarity in structure between the fused and source images, considering luminance, contrast, and structural information. The SF measures the spatial sharpness of the fused image by calculating the row and column frequencies. Moreover, a higher objective index score indicates a better fusion result. The combined use of these metrics allows for a comprehensive and objective assessment of fusion results.

### 4.2. Parameter analysis

In this algorithm, two important parameters are the regularization parameter, *β*, in Eq. (13) and parameter *ψ* in Eq. (15) that controls the window size. In our experiments, 10 pairs of CT-MRI images are selected to determine the settings of these two parameters. Initially, the fixed parameter, *ψ*, is 2. The mean objective evaluation scores of the fused images for *β*∈[2, 3, 4, 5, 6, 7, 8] are displayed in Table 1, where the highest scores for each indicator are bolded. As indicated in Table 1, the scores of Q_MI_, Q_NCIE_, Q_S_, Q_CB_, and SSIM continue to increase as *β* decreases, whereas the scores of AG, SF, and PSNR are reversed. Therefore, considering the comprehensiveness of the performance of the proposed algorithm on each metric, we set the value of the regularization parameter, *β*, to 3.

**Table 1:** Objective evaluation results for different parameter values.

**β**	**Q_MI_**	**Q_NCIE_**	**Q_S_**	**Q_CB_**	**AG**	**SSIM**	**SF**	**PSNR**
2	**0.9086**	**0.8076**	**0.8543**	**0.6576**	9.2653	**0.7375**	39.1295	13.4223
3	0.9008	0.8075	0.8517	0.6560	9.5480	0.7329	40.0730	13.5000
4	0.8942	0.8075	0.8484	0.6542	9.7600	0.7289	40.8056	13.5621
5	0.8883	0.8074	0.8452	0.6524	9.9282	0.7255	41.4098	13.6115
6	0.8823	0.8074	0.8421	0.6510	10.0746	0.7226	41.9536	13.6528
7	0.8771	0.8073	0.8393	0.6495	10.1967	0.7200	42.4087	13.6865
8	0.8721	0.8073	0.8366	0.6480	**10.3015**	0.7177	**42.8184**	**13.7161**

**Figure 5:**
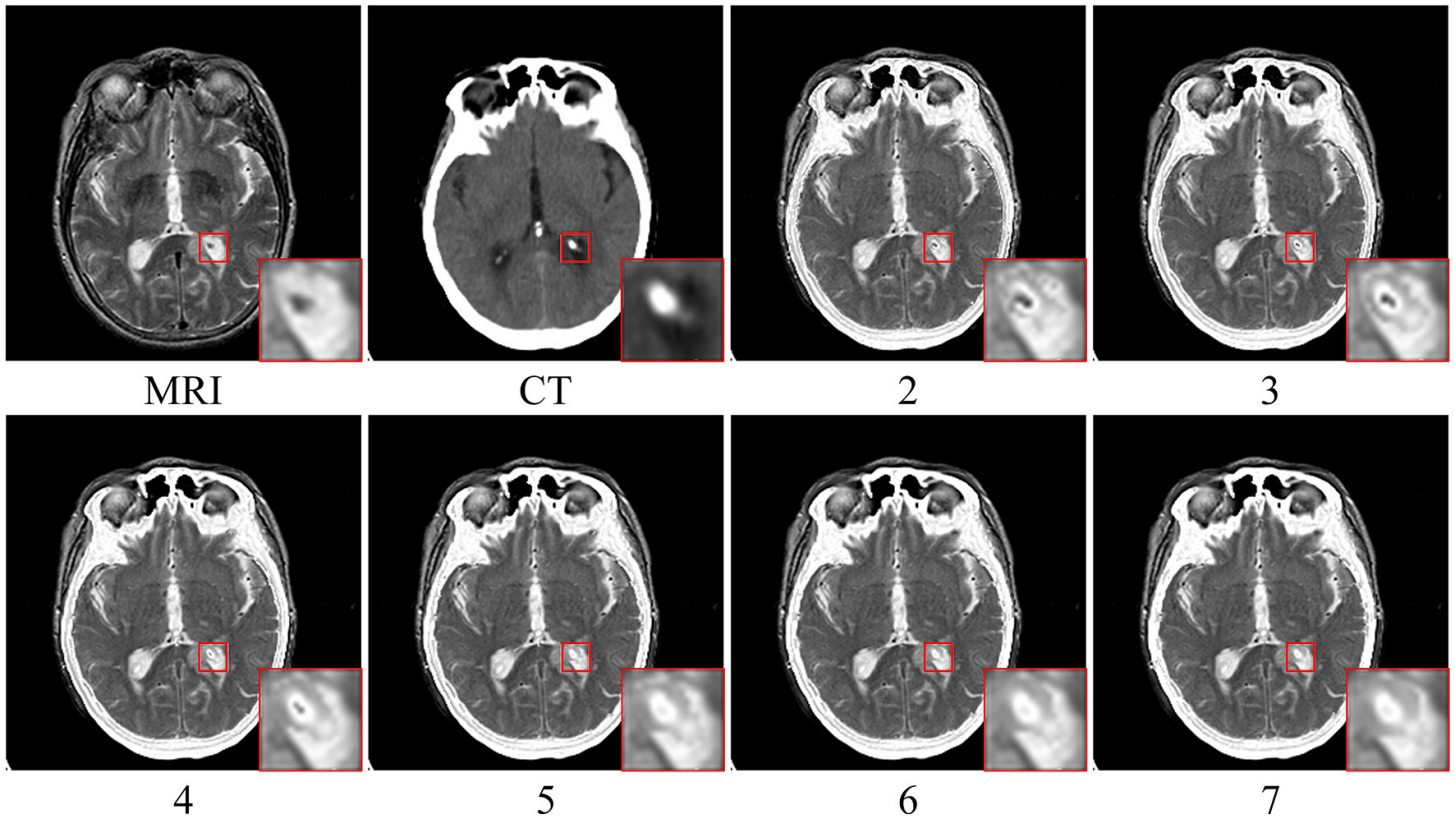
Fusion results corresponding to different values of parameter *ψ*.

The fusion results corresponding to different values of parameter *ψ* when *β* is fixed to 3 are depicted in Figure 5. Upon closer inspection of Figure 5, it becomes apparent that the proposed fusion algorithm suffers from a loss of organ structure information in the source image, particularly in the form of black dots within the white brightness area of the MRI map, as the window size is increased. To obtain a comprehensive understanding of the impact of various parameter values, we conducted a series of quantitative comparison experiments. The mean objective evaluation scores of the fused images across different parameter values of *ψ* are reported in Table 2, revealing that the optimal scores are obtained when *ψ* is set to 3. This parameter value yields superior fusion performance while retaining detailed information in the source image, as supported by both subjective and objective evaluations. Consequently, we set *ψ* to 3 in our proposed algorithm.

**Table 2:** Objective evaluation results for different parameter values.

***ψ***	**Q_MI_**	**Q_NCIE_**	**Q_S_**	**Q_CB_**	**AG**	**SSIM**	**SF**	**PSNR**
2	0.9008	0.8075	0.8517	0.6560	9.5480	0.7329	**40.0730**	**13.5000**
3	0.9071	**0.8075**	0.8565	0.6610	**9.6219**	**0.7338**	40.0225	13.4444
4	0.9074	0.8074	**0.8570**	0.6661	9.4311	0.7336	39.4157	13.3802
5	**0.9078**	0.8074	0.8567	0.6700	9.2148	0.7331	38.9033	13.3183
6	0.9044	0.8073	0.8541	0.6725	8.9324	0.7326	38.4063	13.2695
7	0.9016	0.8072	0.8520	**0.6733**	8.7478	0.7322	38.0730	13.2429

### 4.3. Ablation experiments

We develop a new operator based on the gradient difference and entropy to effectively capture the clear details in the high-frequency components and extract the significant pixel information. We conduct an ablation study to verify whether this method could effectively improve the fusion performance of the proposed algorithm. We randomly selected 20 pairs of source images from the CT-MRI fusion task and compared them with three popular fusion rules based on salient feature measures. These fusion rules replaced the original rule in the proposed algorithm to form new comparison methods (A-ETEM, B-ETEM, and C-ETEM), as described in the following.

#### 4.3.1. A-ETEM

In A-ETEM, we use the energy of Laplacian (EOL) (Huang and Jing, 2007) as a feature extraction algorithm for the high-frequency components. The EOL uses the Laplace operator to analyze the high spatial frequencies that are associated with the sharpness of image boundaries. We replace the original high-frequency fusion rule in the proposed algorithm with the EOL and use it to measure the significant pixel information in the high-frequency component; that is, Eq. (15) is rewritten as


(37) $$\begin{array}{l} GE_{t}\left(i,j\right)=EOL\left(f_{t}^{h}\left(i,j\right),\vartheta \right), \end{array}$$

where *EOL*(·) denotes the EOL detection operator (Huang and Jing, 2007) and ϑ is the size of the Gaussian filter, which we set to 5 in our experiments.

#### 4.3.2. B-ETEM

In B-ETEM, we employ the sum-modified Laplacian (SML) (Li et al., 2022) as a feature extraction algorithm for the high-frequency components; it introduces a modified Laplacian that avoids the cancelation of second-order derivatives with opposite signs in the horizontal and vertical directions. In this algorithm, Eq. (15) is rewritten as


(38) $$\begin{array}{l} GE_{t}\left(i,j\right)=SML\left(f_{t}^{h}\left(i,j\right),\upsilon \right), \end{array}$$

where *SML*(·) represents the SML measurement operator (Li et al., 2022) and υ determines the window size, which is set to 5 in this study.

#### 4.3.3. C-ETEM

The gradient feature can be computed as the first-order directional derivative, quantifying the variation between pixels. In C-ETEM, we use the structure tensor (STO) (Wang and Wang, 2020) as an algorithm for local feature detection to measure the information of pixels with high activity levels in the high-frequency components. That is, Eq. (15) is rewritten as


(39) $$\begin{array}{l} GE_{t}\left(i,j\right)=STO\left(f_{t}^{h}\left(i,j\right)\right), \end{array}$$

where *STO*(·) represents the STO measurement operator (Wang and Wang, 2020).

Table 3 presents the quantitative comparison results of the three methods, where the maximum values of all metric scores are highlighted in bold. The proposed algorithm outperforms the other two methods by achieving the highest scores for five metrics. In the proposed algorithm, we assign a higher weight to clear details compared with structural information with lower pixel values and consider the level of activity of each pixel based on a combination of the gradient difference and entropy values, resulting in the best scores in the information theory-based metrics. Despite not obtaining the highest scores in the AG, SF, and PSNR metrics, the proposed algorithm still achieved a relatively high ranking, securing the second-highest score among all methods. In summary, compared with the current popular image feature detection algorithms, the proposed measurement method exhibits superior performance, effectively improve the accuracy of pixel activity detection, and retains clear high-frequency information.

**Table 3:** Quantitative comparison of ETEM with A-ETEM, B-ETEM, and C-ETEM on CT-MRI fusion task.

**Methods**	**Q_MI_**	**Q_NCIE_**	**Q_S_**	**Q_CB_**	**AG**	**SSIM**	**SF**	**PSNR**
A-ETEM	0.9472	0.8074	0.8652	0.6936	**8.2248**	0.7621	36.5920	13.9159
B-ETEM	0.9328	0.8073	0.8607	0.6929	7.6070	0.7602	34.8062	13.9095
C-ETEM	0.9393	0.8073	0.8575	0.6876	8.1501	0.7599	**36.7950**	**13.9627**
ETEM	**0.9565**	**0.8074**	**0.8662**	**0.6945**	8.1543	**0.7622**	36.6675	13.9414

### 4.4. Analysis of anatomical brain image fusion results

**Figure 6:**
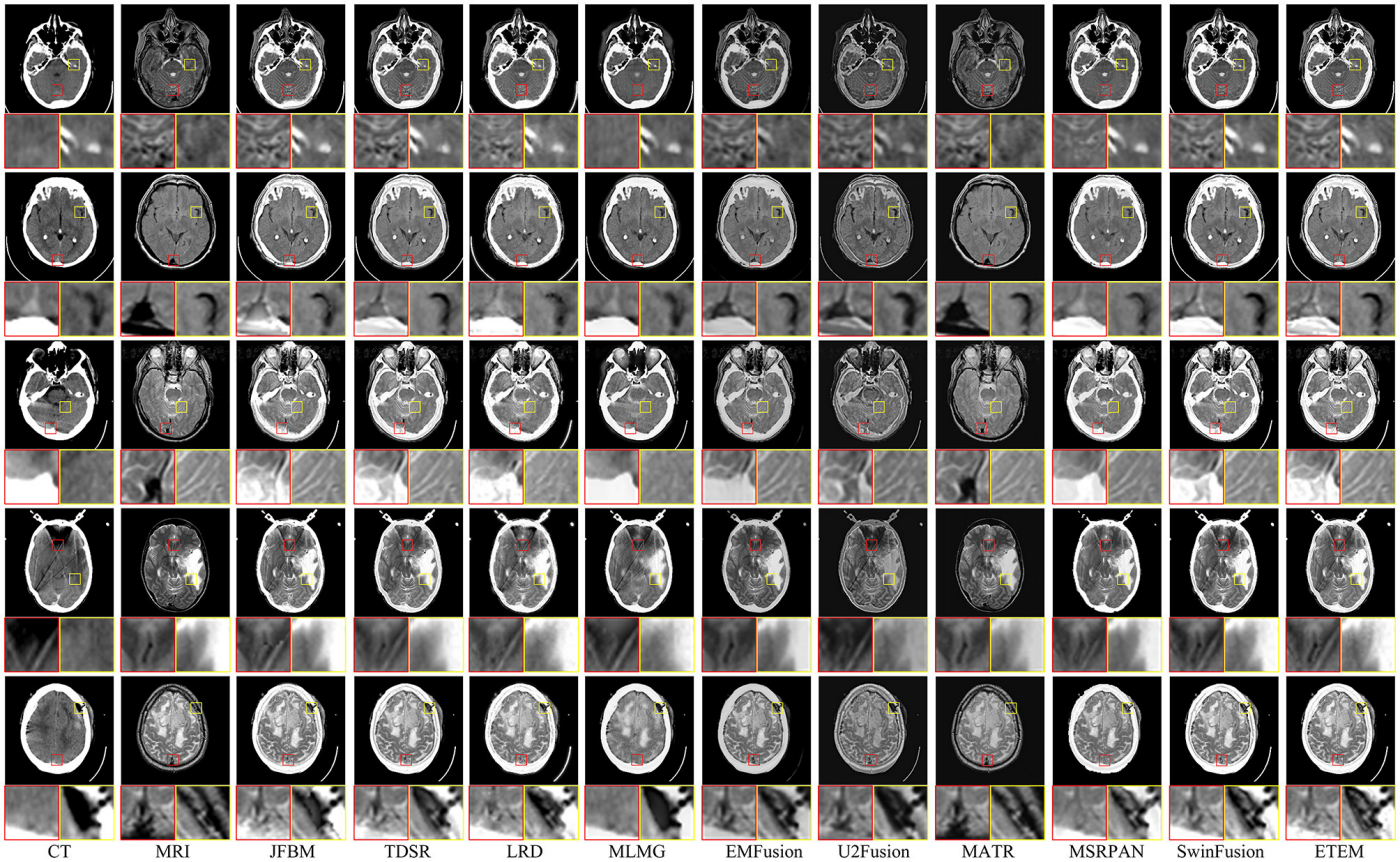
Qualitative comparison of different methods on five CT and MRI image pairs.

Figure 6 shows examples of the fusion of five sets of CT and MRI images, with two local areas enlarged to demonstrate the extent to which the different methods retain texture detail and energy information. Ideally, the fused CT and MRI images should retain the skeletal portion of the CT image and the texture information in the MRI image. As illustrated in Figure 6, JFBM, TDSR, LRD, MLMG, MSRPAN, and SwinFusion methods can extract the skeletal part of the source image effectively and maintain good contrast and illumination. However, residual artifacts at the boundary between the brain tissue and the skull are evident in JBFM, leading to a loss of tissue information in the fusion results. This is because the JBFM method fuses the high and low frequencies of an image through a decision map. Nonetheless, to achieve visually pleasing results for pixels at the boundaries, it may be necessary to combine pixels from different source images. By contrast, the TDSR method can obtain better fusion at the boundary, but the sparse encoding generally has high computational complexity, which makes the method inefficient. Furthermore, the TDSR and MSRPAN methods have a limited ability to identify certain bones with small areas, and some brightness information can be lost. Although the LRD and SwinFusion methods can effectively identify the bone information in the CT images, they have insufficient ability to extract certain fine brain tissue features and texture loss occurs. Although the MLMG method can hardly identify the texture details of brain tissue from the MRI images, it is superior to the aforementioned methods in the extraction of bone luminance information. The EMFusion, U2Fusion, and MATR methods have strong detail perception but perform poorly in maintaining image contrast. For example, the contours in the CT image in Figure 6 are visually white and show the skull, yet all three methods lose plenty of energy information. The ETEM method is salient among its counterparts as it excels in retaining the skeletal luminance information and preserving the intricate texture details of the brain tissue. This notable performance can be attributed to the innovative error texture removal strategy proposed in our approach, which effectively mitigates the loss of intricate details in the fusion process and ensures a balanced and visually appealing contrast in the resulting fused images. In a comprehensive comparison with nine state-of-the-art image fusion methods, the ETEM method emerges as the top performer by effectively preserving and seamlessly integrating the complementary information extracted from multiple source images.

Moreover, to showcase the exceptional capability of the proposed algorithm in preserving the complementary information derived from diverse source images, a comprehensive quantitative comparison is conducted with nine state-of-the-art image fusion techniques, and the results are presented in Table 4. The table features the average scores of all methods for eight evaluation metrics in the CT-MRI fusion task, with the top-performing metric for each method highlighted in bold and the second-best score indicated in red. The proposed algorithm achieves the best scores in three metrics, namely, Q_CB_, AG, and SF, which demonstrate that the proposed algorithm performs the best in retaining source image information and extracting useful features. Moreover, the fused images that are obtained contain more image details and image contrast, as well as higher definition, than those obtained using the comparison methods. Furthermore, the proposed algorithm achieves high scores in the Q_MI_, Q_NCIE_, and Q_S_ metrics. As the proposed algorithm adds an error texture elimination step at the end, some pixel information that is identified as useless in the image may have been lost, resulting in suboptimal performance in the SSIM metric. In summary, the proposed algorithm can achieve better fusion results than the nine state-of-the-art comparison methods for CT-MRI fusion tasks and effectively avoid the loss of details and reduction in sharpness.

**Table 4:** Quantitative comparison of ETEM with nine state-of-the-art methods on three different modal fusion tasks.

**Fusion task**	**Methods**	**Q_MI_**	**Q_NCIE_**	**Q_S_**	**Q_CB_**	**AG**	**SSIM**	**SF**	**PSNR**
CT-MRI	JFBM	0.9277	0.8071	0.8190	0.6773	6.9302	0.7510	31.6202	13.2905
	TDSR	0.7995	0.8064	0.8433	0.6676	6.5686	0.7676	32.1818	13.5705
	LRD	0.7376	0.8058	0.7888	0.6267	6.4320	0.7388	29.3443	13.0585
	MLMG	0.8285	0.8064	0.7617	0.6645	5.5577	0.7521	31.0319	13.6790
	EMFusion	0.7903	0.8063	0.8039	0.6726	4.8595	**0.7737**	19.0622	14.9099
	U2Fusion	0.6663	0.8052	0.3471	0.2970	5.2793	0.2602	19.3021	**15.2920**
	MATR	0.8795	0.8071	0.3099	0.3254	4.8469	0.2135	16.1553	13.0572
	MSRPAN	**1.1591**	**0.8095**	0.7655	0.6482	5.3459	0.6793	26.8027	13.2653
	SwinFusion	0.8469	0.8064	**0.8517**	0.6778	6.8141	0.7162	32.7595	13.2583
	ETEM	0.9577	0.8073	0.8394	**0.6800**	**7.4876**	0.7646	**35.8190**	13.3587
PET-MRI	JFBM	0.9987	**0.8142**	0.9057	**0.6320**	10.8727	0.7091	34.5970	12.4060
	TDSR	0.9112	0.8122	**0.9156**	0.6186	10.5378	0.7137	34.2932	12.4426
	LRD	0.2439	0.8031	0.2452	0.3930	9.0412	0.3180	27.6335	8.3179
	MLMG	0.6687	0.8065	0.7972	0.5993	9.7174	0.6699	33.4689	13.3932
	EMFusion	0.6788	0.8084	0.9044	0.6085	10.0048	**0.7178**	31.7794	**13.4129**
	U2Fusion	0.5727	0.8054	0.3251	0.3554	3.7092	0.2200	11.0752	10.9790
	MATR	07525	0.8116	0.5347	0.3509	9.1313	0.2964	28.0414	13.0674
	MSRPAN	0.9407	0.8113	0.8271	0.5634	10.0005	0.6064	34.7505	12.5030
	SwinFusion	0.6935	0.8099	0.5935	0.3810	**11.4276**	0.3314	**35.0481**	12.1724
	ETEM	**1.0052**	**0.8142**	0.9073	0.6270	10.8472	0.7126	34.6997	12.4044
SPECT-MRI	JFBM	1.0845	**0.8138**	0.9144	**0.6734**	6.5685	0.7317	20.5689	17.7976
	TDSR	1.0050	0.8123	**0.9192**	0.6565	6.3923	0.7338	20.1062	17.8877
	LRD	0.7380	0.8081	0.8859	0.5723	6.2103	0.7099	19.9200	17.2815
	MLMG	0.6789	0.8060	0.8220	0.6109	4.9038	0.7255	15.8330	19.0321
	EMFusion	0.7658	0.8080	0.9081	0.6243	5.5781	**0.7500**	17.7158	**19.1996**
	U2Fusion	0.6648	0.8057	0.3626	0.3200	3.2091	0.2490	10.2598	15.0841
	MATR	0.8156	0.8103	0.5059	0.3350	5.5925	0.2489	17.4157	17.8516
	MSRPAN	1.0519	0.8115	0.8460	0.5739	5.3821	0.5679	18.7914	18.3071
	SwinFusion	0.7904	0.8099	0.5543	0.3745	**6.7181**	0.2892	**21.0548**	17.3384
	ETEM	**1.0921**	0.8136	0.9153	0.6691	6.4476	0.7354	20.3480	17.8147

### 4.5. Analysis of functional brain image fusion results

**Figure 7:**
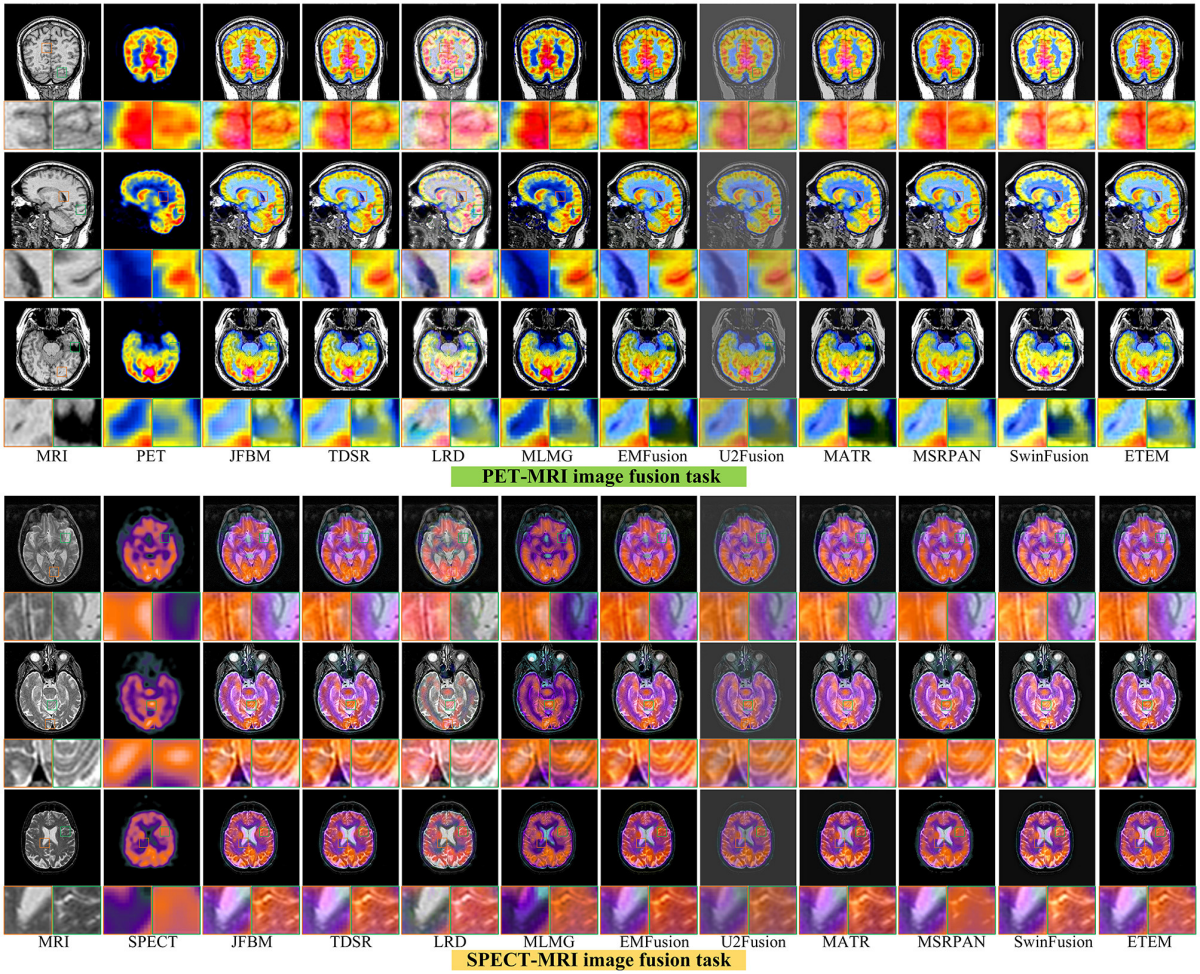
Qualitative comparison of different methods on six functionality image pairs.

Figure 7 illustrates six sets of classical multifunctional brain images and their respective fusion results. We divide the fusion task into the PET-MRI and SPECT-MRI image fusion tasks. As illustrated in Figure 7, relatively satisfactory fusion performance is obtained for all methods. The LRD method exhibits significant color distortion in both fusion tasks owing to its limited ability to capture metabolic information in PET and SPECT. However, it still has a strong ability to extract significant features and retain detailed brain tissue information in MRI maps. The MLMG and MSRPAN methods tend to excessively retain functional information from PET and SPECT images, resulting in the potential loss of textural details in MRI images. This can lead to a reduction in spatial resolution and the omission of crucial information regarding internal brain tissues. Consequently, the accurate visualization of structural information and the detection of soft tissue lesions may be compromised, negatively impacting the physician's ability to make accurate diagnoses and informed decisions. Addressing this limitation is crucial to ensuring the effectiveness of the fusion method in facilitating comprehensive and precise assessments of brain tissue characteristics. The JFBM, TDSR, EMFusion, and MSRPAN methods exhibit superior performance in extracting valuable information from diverse source images while preserving a satisfactory level of contrast. However, despite their strengths, these methods have certain limitations in capturing intricate tissue details and may encounter challenges in preserving fine-grained information, leading to some degree of detailed loss in the fusion results. Although this phenomenon does not appear in the U2Fusion method, the inability of the U2Fusion method to maintain a similar contrast to that of the other algorithms and to extract the skeletal part of the patient would make clinical diagnosis more difficult for physicians. The MATR method exhibits constrained efficacy in extracting luminance information from CT maps, resulting in the loss of certain luminance details. Similarly, the SwinFusion method displays limited sensitivity toward color information, leading to a diminished capacity to interpret the structural characteristics of brain tissue within the fusion results. In summary, the ETEM approach outperforms the state-of-the-art image fusion algorithms in extracting metabolic information from the functional images and tissue information from the MRI images. This tight integration of medical imaging and advanced fusion techniques has the potential to enhance clinical diagnosis, treatment planning, and patient care in various medical disciplines.

Table 4 illustrates the outcomes of our proposed algorithm when compared with nine state-of-the-art image fusion methods on the PET-MRI and SPECT-MRI fusion tasks. These tables contain the average scores of each method in different metrics, providing a comprehensive analysis of their performances. Our algorithm achieved high scores in the Q_MI_, Q_NCIE_, and SF metrics, signifying its capability to extract valuable pixel information from the source image while preserving a superior level of clarity. This indicates the effectiveness of our proposed method in retaining critical details from different modalities. Moreover, the proposed algorithm scores among the highest in the Q_S_, Q_CB_, and AG, which demonstrate that our method can generate visually superior fusion results that identify the significant pixel information in the images. Notably, the JFBM, TDSR, and SwinFusion methods also perform very well in the PET-MRI fusion task. These methods obtain overall high-quality fusion results even if they cannot effectively identify small areas of detailed brain tissue. Table 4 indicates that the proposed algorithm achieves better results for most metrics because it can effectively identify the metabolic and blood flow information in the functional images and effectively extract the brain tissue information in the MRI images. In conclusion, the qualitative analysis indicates that the proposed algorithm can effectively prevent color distortion, residual artifacts, and detail loss while attaining the highest fusion performance compared to the nine state-of-the-art methods.

## 5. Conclusion

This study proposed an MMIF method based on error texture removal. We establish a significant feature extraction operator based on the gradient difference and entropy that can effectively detect the prominent detailed information in the high-frequency subbands. Moreover, we introduce LPC and RW for the fusion of the low-frequency components to detect the pixel information with large energy while preserving the energy regions in the source image. Considering some useful texture information may be distributed in pixels with low energy values, we propose an error texture removal scheme to fuse the texture information using the developed RGF-ILS.

Experiments proved that the proposed method yields better fusion performance than some state-of-the-art methods and can offer comprehensive pathological information and precise diagnosis. The fusion of different modalities of brain images can extract complementary information and facilitate improved visualization and interpretation of brain abnormalities, such as tumors, lesions, and neurodegenerative diseases. Moreover, MBIF technology improves the accuracy and reliability of diagnostic procedures, helping clinicians make informed decisions for treatment planning and monitoring disease progression. However, the proposed method relies on registration and cleans datasets and cannot fuse the unregistered and noise-source images. Therefore, our future study will focus on improving our algorithm to address the unregistered and noise fusion problems and expanding its application to other convergence domains.

## Data availability statement

The original contributions presented in the study are included in the article/supplementary material, further inquiries can be directed to the corresponding author.

## Author contributions

XilL: methodology, software, data curation, writing-original draft, and validation. XiaoSL: conceptualization, formal analysis, writing—review and editing, and funding acquisition. All authors contributed to the article and approved the submitted version.

## Footnotes

^1^http://www.med.harvard.edu/aanlib/home.html.
